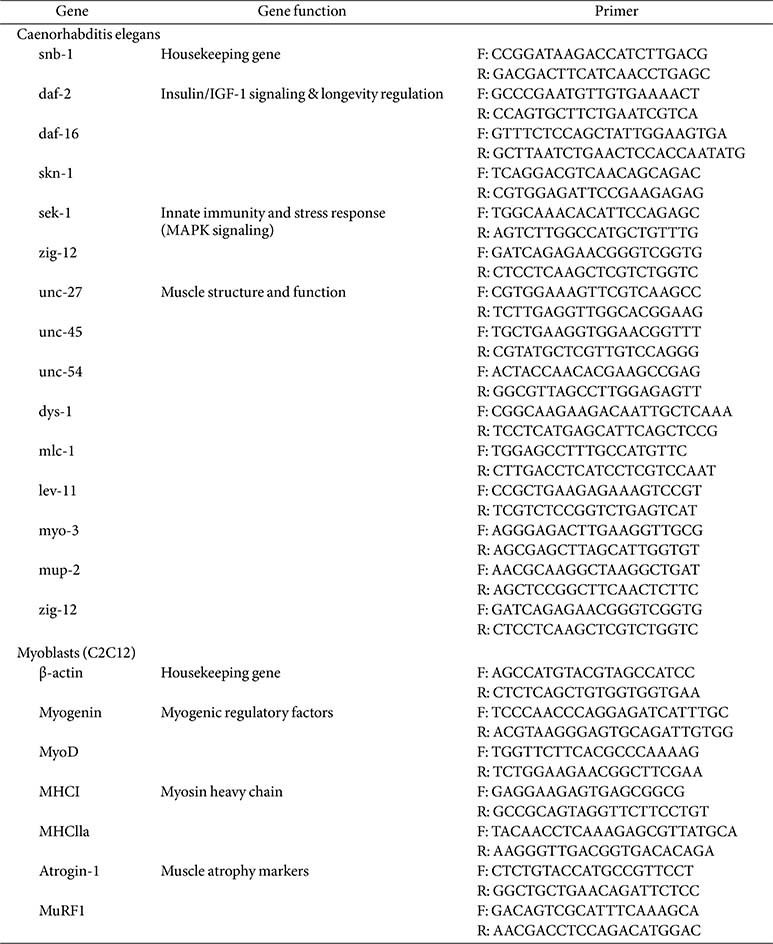# Heat-Treated *Lactiplantibacillus plantarum* KM2 Fermentation Ameliorate Muscular Atrophy

**DOI:** 10.4014/jmb.2506.06042

**Published:** 2025-09-25

**Authors:** Minji Kang, Minkyoung Kang, Moon-Hee Sung, Jong-Hoon Kim, Juyeon Lee, Kwangcheol Casey Jeong, Sangnam Oh

**Affiliations:** 1 Department of Environmental Science and Biotechnology, Jeonju University, Jeonju 55069, Republic of Korea; 2 KookminBio Corporation, Seoul 02826, Republic of Korea; 3 Emerging Pathogens Institute, University of Florida, Gainesville, FL 32611, USA; 4 Department of Animal Sciences, Institute of Food and Agricultural Sciences, University of Florida, Gainesville, FL 32611, USA; 5 Department of Food and Nutrition, Jeonju University, Jeonju 55069, Republic of Korea

**Keywords:** *Lactiplantibacillus plantarum* KM2, postbiotics, sarcopenia, *C. elegans*, cancer cachexia

## Abstract

Sarcopenia, a progressive loss of skeletal muscle mass and function, poses a significant health concern in aging populations and cancer patients. Despite ongoing pharmaceutical research, including drug repurposing strategies, no FDA-approved treatment is currently available for sarcopenia, highlighting the need for safer, food-derived interventions. This study evaluated the anti-aging and muscle-preserving effects of KLP_KM2, a postbiotic formulation derived from *Lactiplantibacillus plantarum* KM2, using *Caenorhabditis elegans* and C2C12 muscle cell models. In *C. elegans*, KLP_KM2 and its components significantly extended lifespan, reduced lipofuscin accumulation, enhanced pharyngeal pumping, and preserved coordinated movement patterns. These effects were accompanied by upregulation of longevity, immune/stress response, and muscle function-related genes. In C2C12 myotubes, KLP_KM2 treatment mitigated CT26-conditioned medium-induced muscle atrophy, restoring myotube diameter and length, increasing expression of myogenic markers (MyoD, myogenin, MHC I, MHC IIa), and downregulating atrophy markers (Atrogin-1, MuRF1). These findings suggest that KLP_KM2 may serve as a promising postbiotic intervention to support muscle health, prevent sarcopenia, and counteract cancer cachexia. Further *in vivo* mammalian studies and clinical trials are warranted to validate its therapeutic potential.

## Introduction

The global transition into an aging society has accelerated, with the proportion of individuals aged 65 years and older surpassing 14% in many countries [1]. This demographic shift has led to an increased need to address aging-related health issues, particularly sarcopenia, a condition defined by the progressive loss of skeletal muscle mass and function [2-4]. Sarcopenia significantly contributes to physical frailty, increased risk of falls, functional dependence, and mortality in the elderly population. Furthermore, muscle wasting is also a hallmark of cancer cachexia, a complex metabolic syndrome characterized by systemic inflammation and unintended weight loss, which further deteriorates patient outcomes [5, 6]. The pathogenesis of sarcopenia and cachexia involves common underlying mechanisms, including dysregulation of protein synthesis and degradation, mitochondrial dysfunction, increased apoptosis of muscle cells, oxidative stress, and chronic low-grade inflammation [7, 8].

These processes ultimately impair muscle regeneration capacity. Several agents are currently under clinical investigation, including selective androgen receptor modulators (SARMs) such as enobosarm (GTx-024)[9], myostatin/activin inhibitors such as bimagrumab [10], and monoclonal antibodies like ponsegromab targeting GDF15 [11]. Although these candidates show promise in enhancing muscle mass or preventing muscle wasting, none have yet received FDA approval. Despite the increasing demand for muscle health improvement, there are currently no established pharmacological therapies, highlighting the urgent need for novel intervention strategies. Thus, safer alternatives derived from food-based bioactive materials are increasingly being explored.

Recent research has focused on natural compounds with anti-inflammatory and antioxidant properties as promising candidates for preventing or mitigating sarcopenia [12, 13]. Phytochemicals such as green tea extract and leonurine have been shown to activate the PI3K/Akt/mTOR pathway, which is critical for protein synthesis and muscle hypertrophy [14, 15]. Concurrently, natural inhibitors of the NF-κB signaling pathway, including olive leaf extract and isobavachalcone, exhibit potent anti-inflammatory effects and contribute to muscle preservation. Moreover, approaches that promote mitochondrial function and inhibit proteolysis have also garnered attention [16, 17].

Among natural sources, soybeans are rich in high-quality proteins and bioactive compounds such as isoflavones, polysaccharides, and soyasaponins, which exhibit notable antioxidative and anti-inflammatory effects that help reduce the risk of diseases such as cancer [18-22]. Previous studies have shown that soybean-derived isoflavones can inhibit muscle atrophy-related pathways while promoting muscle growth and myofiber enlargement [23, 24]. Particularly, Seomoktae, a black soybean variety, has demonstrated efficacy in suppressing muscle atrophy through downregulation of muscle-specific ubiquitin ligases such as MAFbx [25, 26].

Recently, increasing attention has been directed toward the use of probiotics as a therapeutic strategy for mitigating muscle atrophy and sarcopenia. For instance, *Lactiplantibacillus plantarum* TWK10 was shown to enhance skeletal muscle mass and physical performance in aged mice by improving mitochondrial function and reducing oxidative stress [27, 28]. Similarly, *Lacticaseibacillus rhamnosus* IDCC3201 was reported to alleviate muscle atrophy in mice through modulation of microbiome [29].

To ensure safe intake, particularly for the elderly, recent studies have focused on evaluating the efficacy of postbiotics rather than live probiotics. In our previous study, we demonstrated that the culture supernatant of *Lactobacillus rhamnosus* JY02, a strain isolated from kimchi, exerted significant protective effects against dexamethasone-induced muscle atrophy in both *in vitro* and *in vivo* models[30]. Heat-killed *Lacticaseibacillus paracasei* PS23 and its derivatives have been shown to prevent strength loss after muscle injury, improve blood markers of muscle damage and inflammation, and promote recovery with anti-fatigue effects [31].

*L. plantarum* KM2 is a lactic acid bacterium isolated from low-temperature ripened Korean beef [32]. It is a probiotic strain with demonstrated antibacterial and antifungal gene expression, strong intestinal adhesion capacity, and proven safety. Developed in the form of postbiotics material(also referred to as KLP-KM2 or KL-BIOME), this strain has shown beneficial effects on muscle performance, restoration of gut microbial diversity and its safety has also been validated in human applications [33]. Based on these findings, the KM2 strain holds strong potential as a functional material in the fields of food, nutrition, and biotechnology.

This study aimed to investigate the potential of KLP-KM2 in extending lifespan, delaying muscle aging, and improving muscle atrophy in cancer cachexia. We evaluated their efficacy by assessing their ability to ameliorate muscle degeneration in an age-related *in vivo* nematode model and an *in vitro* atrophy model induced by CT26-conditioned medium, thereby establishing their potential as functional agents for muscle health.

## Materials and Methods

### Strain and *C. elegans* Growth Conditions

*C. elegans* strains N2 (Bristol wild-type), PD4251 (ccIs4251), and AY102 (pmk-1(km25)) were used. Worms were cultured and maintained on nematode growth medium (NGM) plates seeded with the standard food source, *E. coli* OP50 by using standard protocol [34]. L1-stage worms were obtained by egg isolation using a sodium hypochlorite–sodium hydroxide solution (Sigma-Aldrich, USA), transferred to NGM plates, and incubated at 25°C. L4-stage worms were used for all experiments.

### Sample Preparation Method

KLP-KM2 and the placebo were manufactured by Kookmin Bio GMP Factory (Republic of Korea). The detailed production process of KLP-KM2 has been previously described in our earlier study [33]. Sample used in this study are summarized in Table 1.

### Longevity and Killing Assay

The lifespan of *C. elegans* was assessed according to previously described methods[35]. Five-fold concentrated OP50 and the sample were diluted at a 1:1 ratio and applied to 35 mm NGM plates. L4-stage N2 or mutant worms were transferred onto the plates using a platinum wire and cultured at 25°C. Survival was monitored daily under a microscope (SZ40, Olympus, Japan).

### Movement and Pharyngeal Pumping Assays

*C. elegans* movement was evaluated following a previously reported method [30]. Synchronized L4-stage worms were exposed to a 1:1 mixture of sample and OP50 for 24 h. Worms were subsequently transferred to fresh sample-containing NGM plates every two days. On days 5, 8, and 10, ten randomly selected worms were placed in M9 buffer, allowed a 10-second adaptation period, and their movement was recorded for 20 sec.

Movements were categorized into three types:

A: S-shaped body movement with pharynx and tail movement,

B: C-shaped movement involving only the anterior body,

C: Limited movement restricted to the pharynx.

Pharyngeal pumping rates were measured on days 5, 8, and 10 after a 10-min adaptation period.

### Fluorescence Microscopy

Strains N2 and PD4251 were used to assess pmk-1 activation and lipofuscin accumulation. Worms were exposed to samples mixed with OP50. On days 1, 5, 8, and 10 post-exposure, worms were mounted on glass slides for fluorescence imaging using a fluorescence microscope (IS 53; Olympus). GFP fluorescence in PD4251 was used to monitor pmk-1 expression, while lipofuscin accumulation was assessed in N2 worms using DAPI (405–488 nm) and TRITC (545/30 nm) filter sets.

### RNA Isolation and Reverse Transcription Quantitative PCR (RT-qPCR)

Total RNA was isolated from *C. elegans* and C2C12 cells (4.0 × 10^4^ cells/well) using QIAzol Lysis Reagent (Qiagen Sciences, USA) and the AccuPrep Universal RNA Extraction Kit (Bioneer, Republic of Korea) according to the previous report [36]. Briefly, cDNA was synthesized using the iScript cDNA Synthesis Kit (Bio-Rad, USA) from RNA samples normalized to 1000 ng. RT-qPCR was performed using Luna Universal qPCR Master Mix (New England Biolabs, USA) on a StepOnePlus real-time PCR system (Applied Biosystems, USA). Gene expression was analyzed using the ΔΔCt method, normalized to the housekeeping gene snb-1 (Table 2).

### Cell Culture and Differentiation

C2C12 mouse myoblasts were cultured in high-glucose DMEM (Welgene Inc., Republic of Korea) supplemented with 10% fetal bovine serum (FBS) and 1% penicillin/streptomycin (P/S, Gibco, Scotland) at 37°C in a humidified incubator with 5% CO_2_ by using a previous report [37]. Differentiation was induced by replacing the growth medium with DMEM containing 2% horse serum (Sigma-Aldrich) and 1% antibiotics when cells reached ~80%confluency. The differentiation medium (DM) was refreshed every two days, and differentiation was continued for six days to allow myotube formation.

### *In vitro* Cancer Cachexia

To induced cachexia-related muscle atrophy, the differentiated myotubes were subsequently treated with CT26-conditioned medium (CM) for 48 h. CT26-CM was prepared for seeding CT26 murine colon carcinoma cells in 100-mm culture dishes at a density of 9.64 × 10^4^ cells/cm^2^. After 24 h, the medium was replaced with serum-free DMEM supplemented with 1% P/S, following a single wash. Cells were then cultured for an additional 4 days under standard culture conditions (37°C, 5% CO_2_). The supernatant was collected and centrifuged at 2,000 ×*g* for 10 min at 4°C to remove cell debris. The CT26-CM was subsequently filtered through a 0.22-μm syringe filter and stored at −80°C until use.

### Cell Viability and Cytotoxicity Assays

C2C12 cells were seeded into 48-well plates and cultured for 24 h. Cells were treated with various concentrations (100–1,000 μg/ml) of KLP-KM2 and CCM for 24 h. Cell viability was assessed using the MTT assay. After MTT reagent (Sigma-Aldrich) incubation for 2 h at 37°C, DMSO was added, and absorbance was measured at 570 nm using a microplate reader (Synergy HT, BioTek Instruments, USA).

### PAS(Periodic Acid-Schiff) Staining and Myotube Diameter Measurement

PAS staining was performed using a kit (VitroVivo Biotech, Media Cybernetics, USA) according to previous report [29]. Cells were washed with cold PBS, fixed with 4% paraformaldehyde, treated with 1% periodic acid, stained with Schiff ’s reagent, and counterstained with Mayer's hematoxylin. Images were captured randomly, and myotube diameters were analyzed using ImageJ software (version 1.42).

### Statistical Analysis

Statistical analysis was performed with GraphPad Prism (version 10.1.2). Differences between groups were assessed using one-way or two-way analysis of variance (ANOVA). Kaplan–Meier survival curves were analyzed using the log-rank test (Mantel–Cox) with Stata software (StataCorp, USA) (StataCorp). All data were obtained from three independent experiments, and statistical significance was set at * *p* < 0.05, ** *p* < 0.001.

## Results

### Effects of *L. plantarum* KM2 Derived Treatments on Lifespan in *C. elegans*

**Fig. 1:**
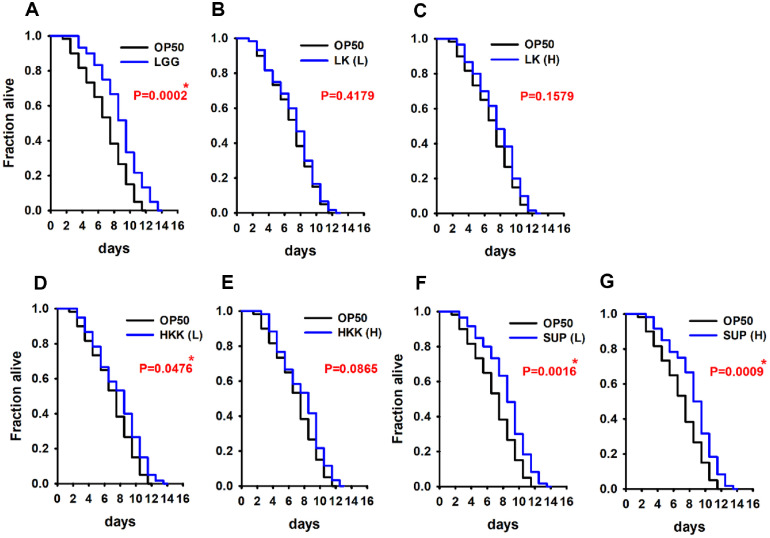
Effect of *L. plantarum* KM2 strains on the lifespan of *C. elegans*. Lifespan assays were performed to assess the effects of *Lactiplantibacillus plantarum* KM2-derived samples on longevity using wild-type N2 strain. The treatment groups included: (**A**) positive control (*Lactobacillus rhamnosus* GG, LGG); (**B–C**) live *L. plantarum* KM2 cells (LK) at low (L) and high (H) doses; (**D–E**) heat-killed *L. plantarum* KM2 cells (HKK) at low (L) and high (H) doses; (**F–G**) cell-free supernatant from *L. plantarum* KM2culture (SUP) at low (L) and high (H) doses. *P < 0.01 vs. OP50 control.

To investigate the lifespan-extending potential of *L. plantarum* KM2, *C. elegans* were treated with live cells (LK), heat-killed cells (HKK), and cell-free culture supernatant (SUP) at low (1 × 10^8^ CFU/ml) and high (1 × 10^9^ CFU/ml) concentrations. A lifespan assay was performed to assess both toxicity and longevity effects. *E. coli* OP50 and *L. rhamnosus* GG (LGG) were used as negative and positive controls, respectively. No toxicity was observed at any concentration of the KM2-derived treatments. Compared with the OP50 control, both LK and HKK treatment groups showed a slight increase in mean lifespan by approximately one day; however, a statistically significant difference was observed only in the low-dose LK group (*p* < 0.05). SUP treatment resulted in an approximate two-day extension of lifespan compared to the negative control, showing similar efficacy to LGG. Notably, both low and high concentrations of SUP exhibited consistent effects without toxicity (Fig. 1). These results suggest that the low dose of heat killed KM2 and culture supernatant of KM2 may contribute to lifespan extension in *C. elegans*, with anti-aging effects.

### Effects of *L. plantarum* KM2 Derived Treatments on Muscle Motility in C.elegans

**Fig. 2:**
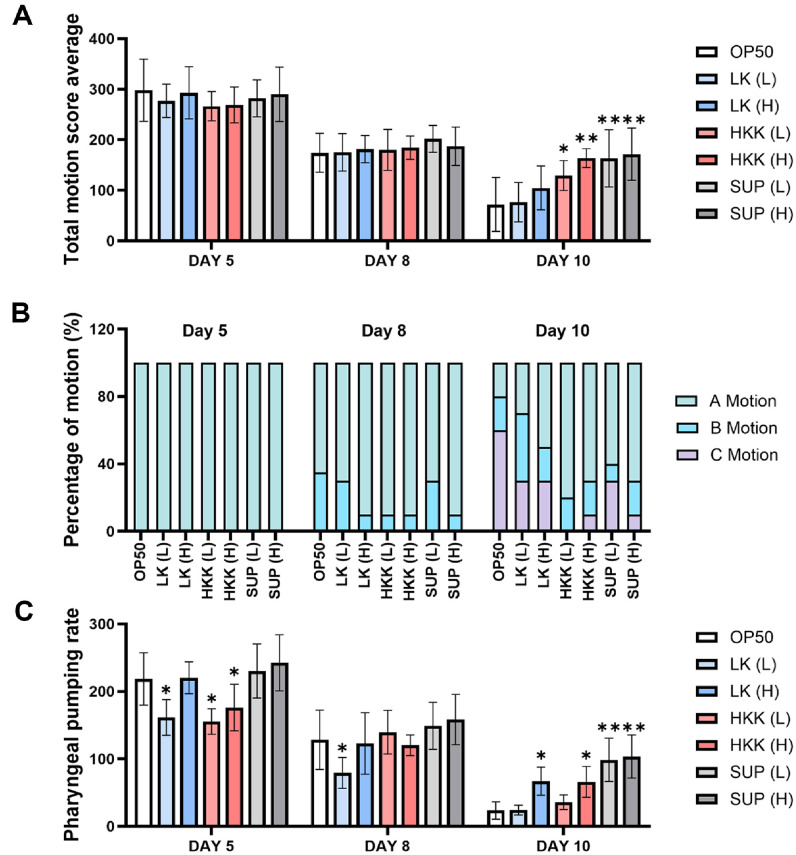
Evaluation of movement and pharyngeal pumping activity in *C. elegans* treated with *L. plantarum* KM2. (**A**) Total movement score of *C. elegans* treated with different forms of *L. plantarum* KM2 in comparison to OP50 control at days 5, 8, and 10. (**B**) Percentage of A, B, and C motion types in treated worms on each day: A-motion (S-shaped fullbody movements), B-motion (C-shaped head or tail movements), and C-motion (pharynx or head-only movements). (**C**) Pharyngeal pumping rates were measured at days 5, 8, and 10. *P < 0.01, **P < 0.001 vs. OP50 control.

To evaluate muscle function of *C. elegans*, both body movement and pharyngeal pumping rates were assessed. Notably, although overall motility decreased from day 5 to day 8, no significant differences were detected among the treatment groups at this time point, suggesting that the reduction was part of a general aging-related decline rather than treatment-specific effects. By day 10, LK (L) and LK (H) exhibited movement levels similar to or slightly higher than OP50, whereas HKK demonstrated superior motility. Notably, the HKK (H) and SUP (H) groups showed significantly improved motility compared to their respective low-dose treatments, and SUP (H) exhibited slightly better motility than SUP (L) (Fig. 2A). The movement patterns of *C. elegans* were categorised into three types (A, B, C), and their proportions were analyzed over time. On day 5, all groups predominantly displayed A motion. By day 8, variations began to emerge, with most groups maintaining A and B motions. By day 10, the OP50 group exhibited a significant increase in C motion (60%), indicating reduced motility. In contrast, other experimental groups showed relatively higher proportions of A and B motions, suggesting improved movement compared to OP50 (Fig. 2B). Pharyngeal pumping rates were also assessed. On days 5 and 8, rates were lower in LK (L) and HKK (L) groups compared to OP50, while LK (H) and HKK (H) showed similar trends. However, by day 10, pharyngeal pumping rates increased in all groups except LK (L). LK (H), HKK (H), SUP (L), and SUP (H) groups demonstrated alleviations in the decline of pharyngeal pumping compared to OP50 (Fig. 2C). These findings suggest that certain treatments, especially high-dose groups, may mitigate reductions in motility and pharyngeal muscle function in *C. elegans*.

### Effects of *L. plantarum* KM2 Derived Treatments on pmk-1 Activation and Lipofuscin Accumulation in *C. elegans*

**Fig. 3:**
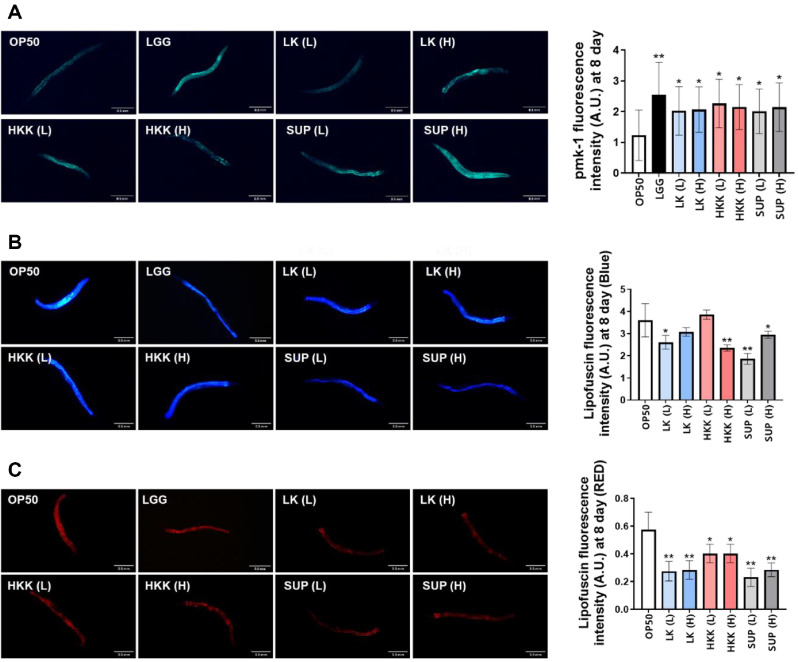
Evaluation of innate immunity and aging markers in *C. elegans* treated with *L. plantarum* KM2. Fluorescence analysis of stress and aging markers in *C. elegans* treated with different forms of *L. plantarum* KM2 on day 10. (**A**) Fluorescence intensity of pmk-1::GFP expressed in the nuclei of body wall muscle cells, representing activation of stress and immune response pathways. Quantitative analysis and representative fluorescence images are shown. (**B**) Blue autofluorescence of lipofuscin, a marker of aging, measured in whole worms. Higher intensity indicates increased accumulation of age-related pigments. Quantification and representative images are included. (**C**) Red fluorescence of lipofuscin assessed as a second marker of aging. Fluorescence intensity values and corresponding representative images are presented. All data were collected on day 10. Treatments include OP50 (control), *P < 0.01, **P < 0.001 vs. OP50 control.

The protein expression level of pmk-1 is a principal indicator of the stress and immune response pathways in *C. elegans* [38]. On day 8, the positive control group (LGG) exhibited the highest expression level, and some KM2-derived samples showed more than a twofold increase in PMK-1 expression compared to the control group (OP50) (Fig. 3A).

Lipofuscin, a well-established aging marker in *C. elegans*, is known to accumulate with age and can be detected through red or blue fluorescence wavelengths. Most KM2-derived treatment groups exhibited a reduction in lipofuscin-associated fluorescence compared to the control. Notably, fluorescence intensity was significantly decreased in the HKK high-dose and SUP low-dose groups, indicating a potential anti-aging effect (Fig. 3B). Red fluorescence detection was more sensitive than blue fluorescence, and a reduction in lipofuscin accumulation was observed across all KM2-derived treatment groups, indicating a broad anti-aging potential (Fig. 3C). Notably, both low and high concentrations of LK and SUP resulted in a marked reduction in lipofuscin accumulation.

### Effects of *L. plantarum* KM2 Derived Treatments on Gene Expression in *C. elegans*

**Fig. 4:**
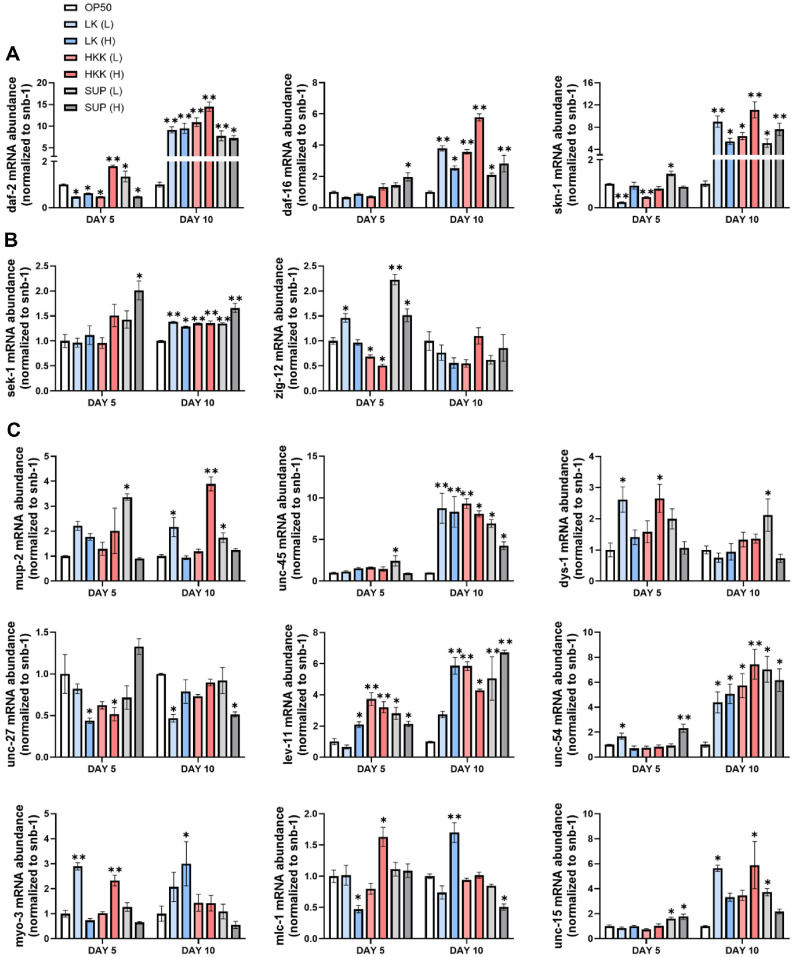
RT-qPCR analysis of immune- and muscle-related gene expression in *C. elegans* treated with different forms of *L. plantarum* KM2. (**A**) Expression of genes involved in insulin/IGF-1 signaling and longevity regulation (daf-2, daf-16, and skn-1); (**B**) innate immunity and stress response via MAPK signaling (sek-1 and zig-12) ; (**C**) muscle structure and function (mup-2, unc45, dys-1, unc-27, lev-11, unc-54, myo-3, mlc-1, and unc-15) all measured in *C. elegans* on days 5 and 10 of treatment. Gene expression was normalized to snb-1 and analyzed using RT-qPCR. *P < 0.05, **P < 0.001 vs. OP50 control.

To investigate the impact of KLP_KM2 component samples on the processes of aging and muscle function, a real time RT-qPCR was performed. Following a five-day period of sample treatment, the expression of six immune-related genes was analyzed. The results demonstrated a significant increase in several immune and antioxidant-related genes, including daf-16, in SUP (L) and SUP (H) (Fig. 4A). It is noteworthy that genes associated with the immune system and the ageing process, including daf-16, sek-1 and age-1, exhibited a marked increase in comparison to the control group (OP50). In the case of daf-2, a significant increase was observed in HKK (H) and SUP (L) (Fig. 4A and 4B). Additionally, alterations in gene expression were identified in genes associated with muscle function. Of particular note were the myosin heavy chain genes (unc-54, myo-3) and tropomyosin-related genes (lev-11 and mup-2), which exhibited a notable increase in expression. These findings suggest that *L. plantarum* KM2 may play a role in maintaining muscle structure and function (Fig. 4C). A comparable trend was identified in the 10-day treatment group. With regard to immune and anti-ageing-related genes, all genes except sek-1 exhibited increased expression, with HKK (H) demonstrating a statistically significant elevation in gene expression. Furthermore, the majority of muscle-related genes demonstrated elevated expression levels in comparison to OP50 (Fig. 4C). These results suggest that *L. plantarum* KM2, particularly HKK (H) and SUP, may positively influence overall sarcopenia alleviation and anti-aging effects by promoting the expression of anti-aging and muscle structure-related genes in *C. elegans*

### Effects of KLP- KM2 and Its Components on Lifespan in *C. elegans*

**Fig. 5:**
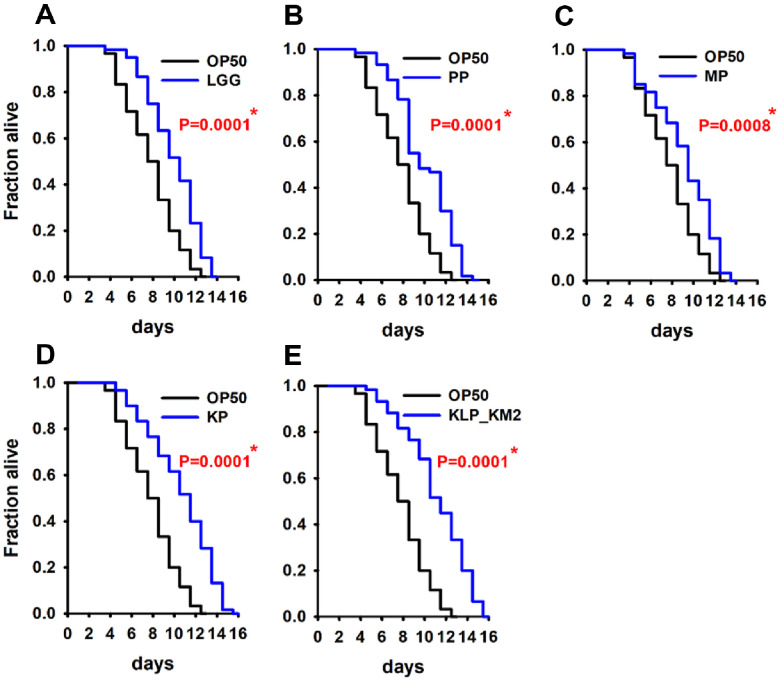
Effect of KLP_KM2 and its components on the lifespan of *C. elegans*. Lifespan assays were performed to assess the longevity-promoting effects of KLP_KM2 and its constituent samples using the wild-type N2 strain of *C. elegans*. The treatment groups included: (**A**) positive control (*Lactobacillus rhamnosus* GG, LGG); (**B**) puffed Socheongja powder (PP); (**C**) *Lactobacillus* medium powder (MP); (**D**) *L. plantarum* KM2-derived supernatant powder (KP); (E) KLP_KM2 powder. *P < 0.05 vs. OP50 control.

In the present study, the anti-aging and muscle health promoting potential of the HKK and SUP samples was evaluated, leading to the development of a postbiotic formulation named KLP_KM2. The manufacturing process of KLP_KM2 was previously described in an earlier study [33], and for the current study, its representative components were categorized into puffed soybean powder (PP), culture medium powder (MP), *L. plantarum* KM2 supernatant powder (KP) (Table 1). These individual treatments including LGG as a positive control, along with KLP_KM2, were evaluated for their effects on longevity and muscle function. In comparison to OP50 as a normal feed, All samples, including KLP_KM2, exhibited increased longevity (Fig. 5A-5E). It is noteworthy that the PP, KP, and KLP_KM2 samples exhibited higher lifespan than the positive control, LGG. These findings indicate that KLP-KM2 and its components may have a anti-aging effect on lifespan extension in *C. elegans* (Fig. 5B, 5C, and 5E).

### Effects of KLP- KM2 and Its Components on Muscle Function in *C. elegans*

**Fig. 6:**
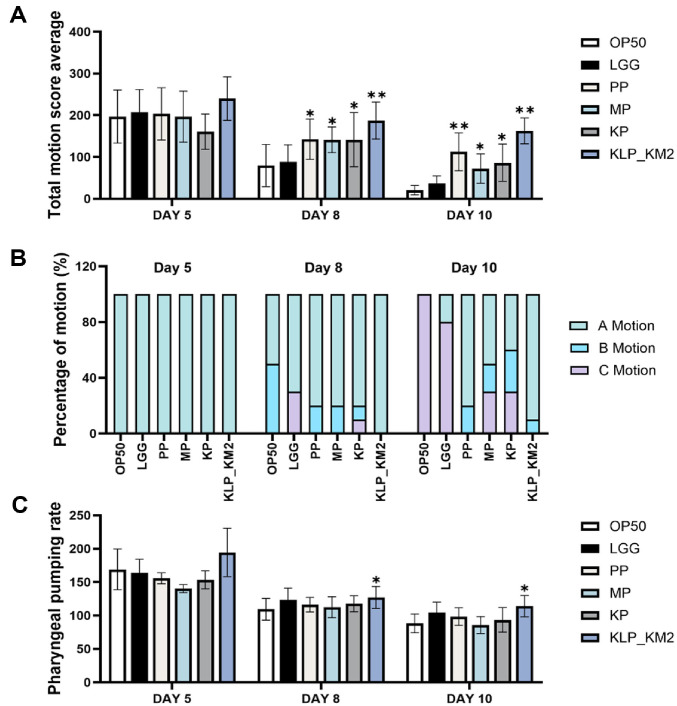
Evaluation of movement and pharyngeal pumping activity in *C. elegans* treated with KLP_KM2 and its components. (**A**) Total movement score of *C. elegans* treated with KLP_KM2 and its components (LGG, PP, MP, KP) in comparison to OP50 control at days 5, 8, and 10. (**B**) Percentage of A, B, and C motion types in treated worms on each day: Amotion (S-shaped full-body movements), B-motion (C-shaped head or tail movements), and C-motion (pharynx or head-only movements). (**C**) Pharyngeal pumping rates were measured at days 5, 8, and 10. *P* < 0.05, *P* < 0.001 vs. OP50 control.

To evaluate the effects of KM2-derived samples on muscle function in *C. elegans*, body movement and pharyngeal pumping were analyzed on days 5, 8, and 10 using ten worms per group at each time point. No significant differences in total motion scores were observed on day 5. On day 8, PP, KP, and KLP_KM2 showed significantly higher motility compared to OP50. By day 10, OP50-treated worms exhibited markedly reduced movement, whereas PP, KP, and KLP_KM2 maintained significantly higher scores (Fig. 6A). The movement types of *C. elegans* were classified into A (normal), B (reduced anterior), and C (pharyngeal-only) motion patterns. On day 5, all groups predominantly exhibited A-type motion. By day 8, the proportion of B- and C-type motions increased in the OP50 group, whereas the KM2-derived groups, especially KLP_KM2, maintained a higher percentage of A-type motion. On day 10, PP and KLP_KM2 treatment groups retained 80% and 90% A-type motion, respectively, suggesting preservation of coordinated movement (Fig. 6B). pharyngeal pumping rates showed no differences on day 5. On day 8 and 10, KLP_KM2 showed significantly higher pumping rates than other treatment (Fig. 6C). These results suggest that KM2-derived samples, particularly KLP_KM2, may help preserve both locomotor activity and pharyngeal muscle function in aged *C. elegans*.

### Evaluation of pmk-1 Activation and Lipofuscin Accumulation by KLP_KM2 and Its Components in *C. elegans*

**Fig. 7:**
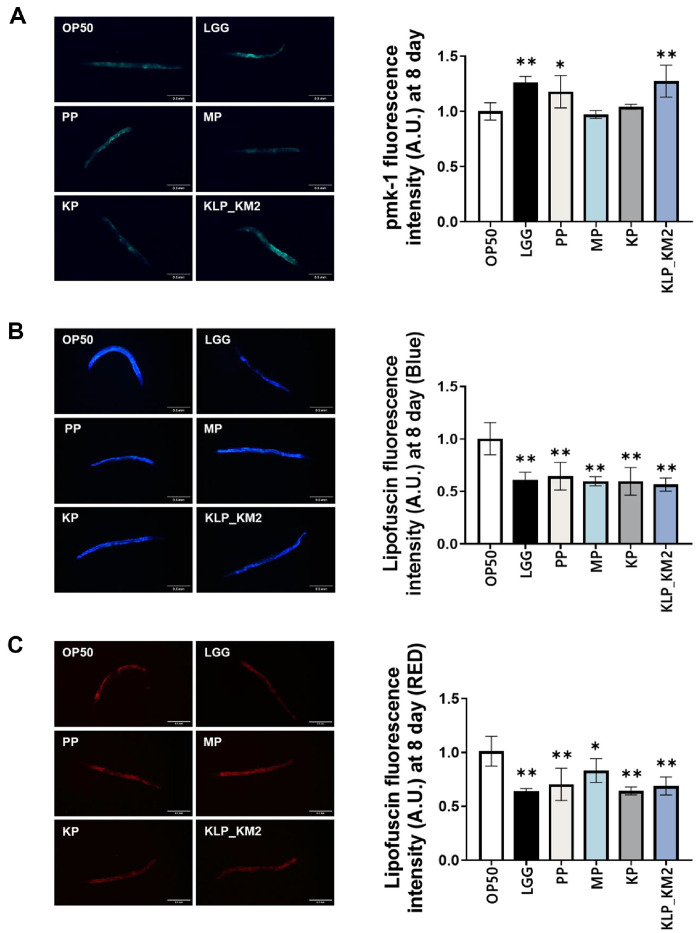
Fluorescence intensity and aging markers in *C. elegans* treated with KLP_KM2 and its components. (**A**) Fluorescence intensity of transgenic *C. elegans* expressing GFP in the nuclei of body wall muscle, specifically the PMK-1 gene, treated with KLP_KM2 and its components at day 10. (**B**) Blue autofluorescence of lipofuscin, an aging marker, measured in treated worms. (**C**) Red autofluorescence of lipofuscin *P* < 0.05, *P* < 0.001 vs. OP50 control.

To evaluate the immune-related and anti-aging effects of KM2-derived samples, pmk-1 expression and lipofuscin fluorescence were analyzed in *C. elegans*. On day 8, KLP_KM2 and PP showed significantly higher pmk-1 expression compared to the OP50 control, with levels comparable to the positive control, LGG (Fig. 7A). Blue and red autofluorescence of lipofuscin, a marker of aging were reduced in *C. elegans* fed with all components compared to OP50, indicating a potential delay in lipofuscin accumulation and slow aging process (Fig. 7B and 7C).

### Gene Expression Analysis of KLP_KM2 and Its Components in *C. elegans*

To investigate the gene expression changes associated with anti-aging and muscle function, *C. elegans* were treated with KLP_KM2 and its components, and samples were collected on days 5 and 10 for analysis. Genes related to the insulin/IGF-1 signaling and longevity pathways, including daf-2 were upregulated in all treatment groups compared to the OP50 control on day 5. Although the expression levels of these genes declined by day 10, daf-16 and skn-1 remained elevated in the PP and KLP_KM2 groups, indicating a sustained expression.

**Fig. 8:**
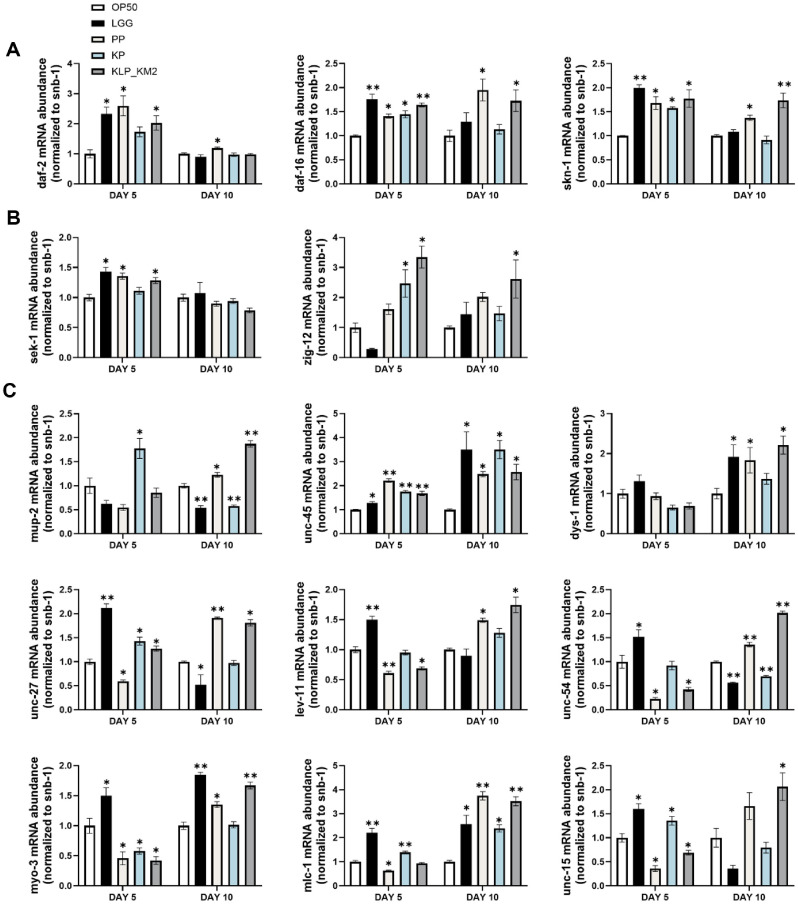
RT-qPCR analysis of immune- and muscle-related gene expression in *C. elegans* treated with KLPKM2 and its components. (**A**) Insulin/IGF-1 signaling and longevity regulation in *C. elegans* on days 5 and 10 following treatment with PP, MP, KP, and KLP_KM2. (**B**) Innate immunity and stress response (MAPK signaling) in *C. elegans* on days 5 and 10 of treatment. (**C**) Muscle structure and function in *C. elegans* on days 5 and 10 of treatment. Gene expression was normalized to snb-1 and analyzed using RT-qPCR. *P < 0.05, **P < 0.001 vs. OP50 control.

For innate immunity and stress response pathways, increased expression of sek-1 and zig-12 was observed on day 5, and notably, zig-12 expression remained elevated in the KLP_KM2 group through day 10. Among nine genes associated with muscle structure and function, a general upregulation was detected in response to KLP_KM2 and its components, with more genes showing increased expression on day 10 than on day 5. Significant upregulation of muscle-related genes was particularly evident in the PP and KLP_KM2 treatment groups (Fig. 8C).

Collectively, these findings suggest that long-term administration of KLP_KM2 may effectively activate anti-aging and immune responses, while promoting the expression of key muscle-related genes, thereby contributing to the alleviation of sarcopenia.

### Inhibitory Effects of KLP_KM2 and Its Components on Muscle Atrophy *In Vitro* Cancer Cachexia

**Fig. 9:**
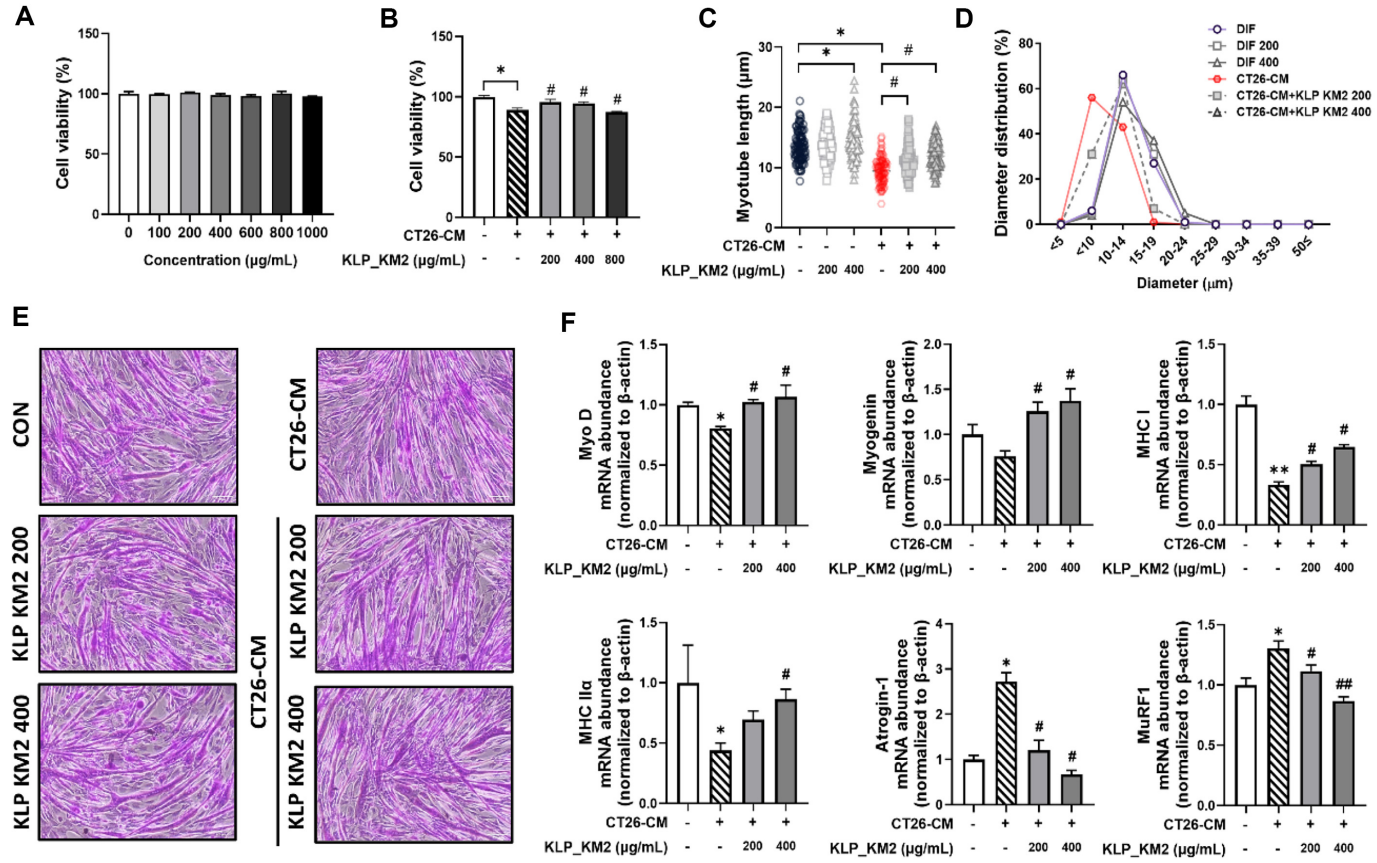
Evaluation of the effects of KLP-KM2 on C2C12 myoblast cells and CT26-CM-induced muscle atrophy. (**A**) Cell viability of C2C12 myoblasts treated with various concentrations of KLP-KM2 (100–1,000 μg/ml). (**B**) KLP-KM2 treatment at 200 and 400 μg/ml on cell viability in dexamethasoneinduced muscle atrophy. (**C**) Myotube length of C2C12 cells after CT26-CM treatment and KLP-KM2 exposure at 200 and 400 μg/ml. (**D**) Distribution of myotube diameter in C2C12 cells treated with CT26-CM to induce muscle atrophy and KLP-KM2 at 200 and 400 μg/ml (**E**) PAS staining images of C2C12 myotubes after CT26-CM treatment and KLP-KM2 exposure (200 and 400 μg/ml). (**F**) RT-qPCR analysis of mRNA expression levels of muscle-related genes (Myogenin, MHC I, MyoD, MHC IIa, Atrogin-1, MuRF1) in C2C12 cells treated with KLP-KM2. #*P* < 0.05 vs. CON, ##*P* < 0.001 vs. CON, *P < 0.05 vs. CCM, **P < 0.001 vs. CCM. Each assay was independently repeated three times to ensure reproducibility and statistical reliability. Data represent the mean ± SD of three independent experiments.

To further validate the muscle-protective effects of KLP_KM2 observed in *C. elegans*, a cancer cachexia model was established using C2C12 myotubes. This model was used to assess whether KLP_KM2 could ameliorate muscle atrophy under pathological conditions. To evaluate the cytotoxicity and potential efficacy of KLP_KM2, C2C12 myotubes were treated with various concentrations (100-1,000 μg/ml) of KLP_KM2 for 24 h. No cytotoxicity was observed at any concentration (Fig. 9A). In cancer cachexia conditions, C2C12 myotubes were treated with CT26-conditioned medium (CCM), after which recovery effects were assessed by treatment with KLP_KM2. concentrations of 200 and 400 μg/ml recovered cell viability and were selected for subsequent experiments (Fig. 9B).

Analysis of myotube diameter and length revealed a significant reduction in the CCM-treated group, whereas KLP_KM2 treatment led to a dose-dependent increase in both parameters (myotube length and diameter distribution). In particular, treatment with 400 μg/ml of KLP_KM2 showed the most pronounced recovery, suggesting inhibition of muscle atrophy (Fig. 9C and 9D).

PAS staining supported these findings, showing that myotubes in the CCM group were thin and irregular, whereas those in the KLP_KM2-treated group were thicker and more intact, indicating enhanced myotube formation (Fig. 9E). The expression levels of muscle differentiation markers - Myogenin, MHC I, MyoD, and MHC IIa were significantly increased following KLP_KM2 treatment, particularly at 400 μg/ml. In contrast, the expression of these genes was suppressed in the CCM-only group. Conversely, the expression of atrophy-related genes Atrogin-1 and MuRF-1 was markedly elevated in the CCM group, but significantly suppressed by KLP_KM2 co-treatment in a dose-dependent manner (Fig. 9F).

These findings suggest that KLP_KM2 not only protects against cancer cachexia-induced muscle atrophy but also promotes muscle regeneration by modulating myogenic and atrophic gene expression.

## Discussion

With the global acceleration of population aging, sarcopenia has emerged as a critical public health concern due to its association with reduced mobility, frailty, and mortality [13]. While current therapeutic candidates including SARMs, myostatin inhibitors, and anti-inflammatory agents have shown potential, no pharmacological treatment has yet received FDA approval. Recent efforts to identify pharmacological treatments for sarcopenia have increasingly focused on drug repurposing strategies, given that no agent has yet received FDA approval specifically for sarcopenia management [39].

However, this underscores the need for safer, food-derived interventions that can promote muscle health, particularly in older adults and cancer patients, whose incidence increase with age. In this study, we evaluated the functional efficacy of KLP_KM2, a postbiotic formulation derived from *L. plantarum* KM2, using both *C. elegans* and C2C12 cell models. *C. elegans* was chosen for its well-defined genetic pathways regulating aging, neuromuscular function, and lifespan, making it a valuable model for anti-aging and muscle function studies [40]. C2C12 myotubes were selected for their established relevance in muscle differentiation and atrophy research, particularly involving PI3K/Akt/mTOR, FoxO, and AMPK pathways [41]. Our findings demonstrate that KLP_KM2 and its constituent components (SUP, PP, KP) significantly extended lifespan, improved motility, and attenuated age-related physiological decline in *C. elegans* (Figs. 1-3). Especially, the increase in pharyngeal pumping in *C. elegans* indicates better neuromuscular maintenance, since diminished pumping is associated with structural and functional deterioration of the pharynx [42].

These effects were accompanied by the upregulation of genes associated with insulin/IGF-1 signaling (daf-2, daf-16), immune and stress responses (pmk-1, sek-1), and muscle function (myo-3, unc-54, zig-12), consistent with previous studies showing similar structural gene expression increases in exercise models [43]. These observations suggest that KLP_KM2 activates multiple regulatory pathways related to longevity and muscle maintenance. Moreover, fluorescence imaging revealed that KLP_KM2-treated worms exhibited reduced lipofuscin accumulation, a hallmark of cellular aging, further supporting its anti-aging potential. The sustained improvement in pharyngeal pumping rates and preservation of A-type movement also reflect its capacity to mitigate neuromuscular decline associated with aging.

To explore the translational relevance of these findings, we employed a C2C12-based cancer cachexia model induced by CT26-conditioned medium. CCM, derived from colon cancer cells, contains inflammatory cytokines and catabolic factors, including NF-κB, IL-6, and TGF-β, which promote protein degradation pathways in muscle cells while inhibiting protein synthesis signals, ultimately leading to muscle atrophy [44]. KLP_KM2 treatment significantly restored myotube diameter and length, as confirmed by morphological analysis and PAS staining. Additionally, RT-qPCR revealed increased expression of muscle differentiation markers (Myogenin, MyoD, MHC I, MHC IIa) and reduced expression of atrophy-related markers (Atrogin-1, MuRF1). These results align with previous reports of postbiotics substances modulating myogenesis and inhibiting catabolic signaling via the Akt/FoxO3a/mTOR pathway [45, 46]. Postbiotics consumed as dietary supplements have been reported to beneficially influence key physiological processes, such as inflammatory responses, immune function [47], energy metabolism, and insulin sensitivity[48, 49]. Future studies should investigate whether KLP_KM2 can similarly enhance these systemic functions, providing additional evidence for its potential as a functional food ingredient for aging-related health management.

Together, these results suggest that KLP_KM2 exerts multifaceted effects, including lifespan extension, anti-sarcopenic activity, and muscle regeneration, via modulation of immune, metabolic, and muscle-specific gene expression. Notably, the use of heat-killed *L. plantarum* KM2 may offer a safer and more stable alternative to live probiotics, which can pose risks in immunocompromised individuals. While the *C. elegans* model provides valuable insights into aging, muscle regulation, and gene expression, its direct applicability to human physiology is limited. Nonetheless, this study is meaningful in that we employed KLP_KM2, a substance already verified for safety in humans[33], to demonstrate its anti-aging and muscle-protective effects in *C. elegans*. Although the safety of KLP_KM2 has been previously confirmed in human studies, this investigation did not include clinical trials targeting older adults who are otherwise healthy but meet diagnostic criteria for sarcopenia due to significant muscle loss. Future human intervention studies assessing muscle mass, grip strength, and physical performance are warranted to validate the clinical efficacy of KLP_KM2 in preventing or mitigating sarcopenia. While the effects of KLP_KM2 are clearly demonstrated in *C. elegans* and C2C12 cell models, their practical applicability in humans is further supported by evidence from clinical studies. For example, a randomized, placebo-controlled trial in older adults showed that daily supplementation with heat-killed *Lacticaseibacillus paracasei* PS23 (2 × 10^10^ cells/day) for 12 weeks significantly improved lower-limb muscle strength, reduced proinflammatory markers (*e.g.*, IL-6, CRP), and increased serum testosterone levels [31]. Similarly, a 6-week double-blind, placebo-controlled trial with heat-killed *L. plantarum* TWK10 (3×10^11^ cells/day) demonstrated significant improvements in muscle endurance, grip strength, and muscle weight while reducing fatigue-related biomarkers in healthy male adults [27, 50]. These findings are consistent with our previously reported human-safe dose of heat-killed L. plantarum KM2 (5 × 10^9^ cells/day) [33]. Therefore, KLP_KM2, used at comparable or higher concentrations, has potential to support muscle health and mitigate sarcopenia in aging or cachexia-related conditions. Thus, clinical trials using this range of doses may help confirm its therapeutic potential for improving muscle health and alleviating cancer cachexia in human populations. As the current *in vitro* model of cachexia lacks systemic complexity, future studies employing *in vivo* mammalian models will be essential to elucidate the mechanistic pathways, particularly those involving the gut-muscle axis. Research in cancer cachexia patients, in particular, should be conducted with heightened clinical precision, as this population may present unique physiological vulnerabilities. While KLP_KM2 has shown a favorable safety profile in prior human applications, its safety and efficacy must be independently verified in cancer patients to ensure clinical applicability and minimize potential risks. To elucidate the underlying mechanisms of the gut–muscle axis, future studies will incorporate multi-omics approaches, including metagenomics and metabolomics, in murine models, along with fecal microbiota transplantation (FMT) in germ-free mice. Therefore further research in cancer cachexia should aim to deepen understanding of the gut microbiota’s role and identify effective microbial targets, ultimately translating these insights into clinically applicable interventions[49].

## Conclusion

**Fig. 10:**
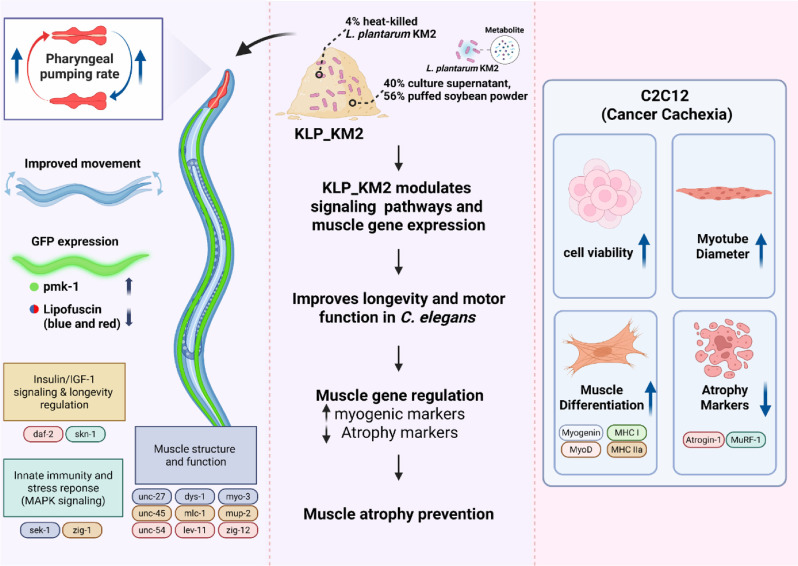
Schematic overview of the beneficial effects of KLP_KM2, a postbiotic formulation composed of heatkilled *Lactiplantibacillus plantarum* KM2. In *C. elegans*, KLP_KM2 improved lifespan and motor function by modulating pharyngeal pumping rate, reducing lipofuscin accumulation, and upregulating genes associated with longevity, immunity, and muscle function. In a cancer cachexia-induced C2C12 model, KLP_KM2 enhanced cell viability and myotube diameter, promoted muscle differentiation, and downregulated muscle atrophy markers, suggesting its potential for sarcopenia management.

This study evaluated the efficacy of a postbiotic formulation derived from *L. plantarum* KM2 fermentation (KLP_KM2), developed to improve muscle health. In *C. elegans*, KLP_KM2 significantly prolonged lifespan, improved motility, and exerted anti-aging effects. These benefits were accompanied by the upregulation of genes related to longevity, immune/stress response, and muscle function. In a cancer cachexia-induced C2C12 myotube atrophy model, KLP_KM2 treatment led to increased myotube thickness and reduced the expression of atrophy-related gene markers and increased muscle differentiation markers (Fig. 10). These results suggest that KLP_KM2 may attenuate muscle degradation while promoting muscle regeneration and support the potential application of KLP_KM2 as a functional postbiotic agent for enhancing muscle health and mitigating sarcopenia in aging populations and cancer patients. To further verify these findings, future clinical studies should evaluate the efficacy and optimal dosage of KLP_KM2 in humans, considering individual differences in gut microbiota and metabolic responses.

## Figures and Tables

**Table 1:** Experimental samples: Controls and *Lactiplantibacillus plantarum* KM2 9reparations.

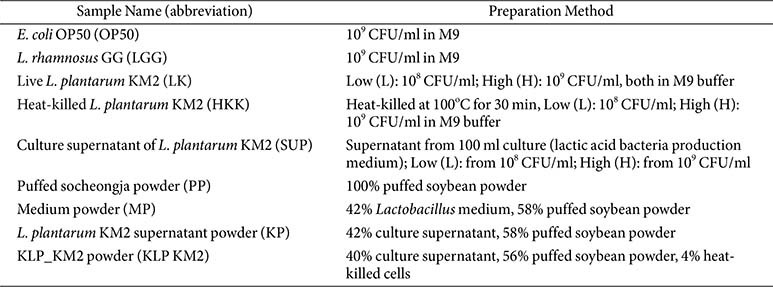

**Table 2:** Primer sequences of target genes for qRT-PCR.